# Voltage-gated sodium channels from the bees *Apis mellifera* and *Bombus terrestris* are differentially modulated by pyrethroid insecticides

**DOI:** 10.1038/s41598-018-37278-z

**Published:** 2019-01-31

**Authors:** Aklesso Kadala, Mercédès Charreton, Pierre Charnet, Thierry Cens, Mathieu Rousset, Mohamed Chahine, Bernard E. Vaissière, Claude Collet

**Affiliations:** 1 INRA, UR 406 Abeilles et Environnement, 84914 Avignon, France; 2 UMT PRADE, Protection des Abeilles dans l’Environnement, 84914 Avignon, France; 3 CNRS, UMR 5237, Centre de Recherche de Biochimie Macromoléculaire, Université Montpellier 2, Montpellier, France; 4 Department of Medicine, Université Laval, Quebec City, QC G1K 7P4 Canada

## Abstract

Recent experimental and in-field evidence of the deleterious effects of insecticides on the domestic honey bee *Apis mellifera* have led to a tightening of the risk assessment requirements of these products, and now more attention is being paid to their sublethal effects on other bee species. In addition to traditional tests, *in vitro* and *in silico* approaches may become essential tools for a comprehensive understanding of the impact of insecticides on bee species. Here we present a study in which electrophysiology and a Markovian multi-state modelling of the voltage-gated sodium channel were used to measure the susceptibility of the antennal lobe neurons from *Apis mellifera* and *Bombus terrestris*, to the pyrethroids tetramethrin and esfenvalerate. Voltage-gated sodium channels from *Apis mellifera* and *Bombus terrestris* are differentially sensitive to pyrethroids. In both bee species, the level of neuronal activity played an important role in their relative sensitivity to pyrethroids. This work supports the notion that honey bees cannot unequivocally be considered as a surrogate for other bee species in assessing their neuronal susceptibility to insecticides.

## Introduction

Pyrethroids are a large class of neurotoxic insecticides introduced in the 1970s for plant protection and public health purposes. After decades during which risk assessment focused mainly on the mortality rates of exposed organisms^1–5^, the sublethal effects of these compounds on non-target organisms are now being considered. To date, most of the effort have focused on the analysis of the sublethal effects of pyrethroids on the domestic honey bee *Apis mellifera* (*Am*) at the individual^6–9^, tissue and cellular levels^10–14^. Only a few data are available for bee species other than *Am*, including Bombus terrestris (Bt)^15–17^.

**Figure 1:**
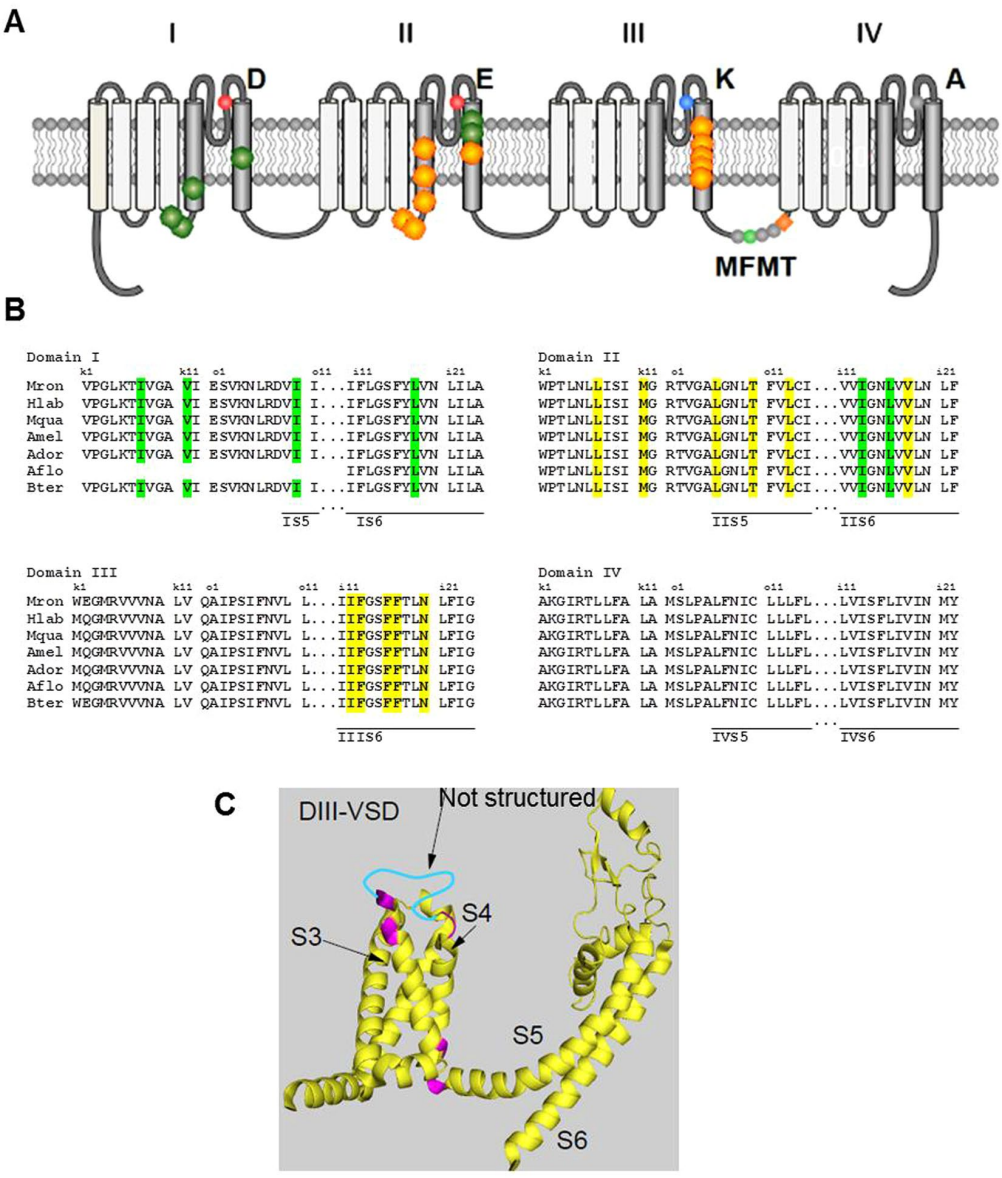
Voltage-gated sodium channels from several bee species. (**A**) The alpha subunit of the voltage-gated sodium channel has four transmembrane domains (I to IV) and each domain is made of six segments (S1–S6). Pyrethroids can interact with the voltage-gated sodium channel in sites highlighted in orange (site 1) and green (site 2) colours. Amino acids residues DEKA form the inner ring of the channel’s selectivity filter and the MFMT pattern is part of the inactivation gate of the channel. (**B**) Multiple amino acid sequences alignment of the voltage-gated sodium channel alpha subunit from seven bee species. Sequences are from *Megachile rotundata* (Mrot, accession number XP_012144116), *Habropoda laboriosa* (Hlab, ENA_KOC69810), *Melipona quadrifasciata* (Mqua, ENA_KOX71756), *Apis mellifera* (Amel, AMB38675), *Apis dorsata* (Ador, XP_006613070), *Apis florea* (Aflor, XP_012347667), *Bombus terrestris* (*Bter*, XP_012167116). Putative sites of interaction for pyrethroids are highlighted in yellow (site 1) and green (site 2). Binding sites for pyrethroids show a remarkable conservation across the species. (**C**) Three-dimensional representation of the domain III of the Na_V_. Differences in amino acids sequences between *Am* and *Bt* are highlighted in magenta.

Recent evolution in toxicology now focuses on the molecular modes of action of insecticides in order to better anticipate their sublethal effects. The primary molecular targets of pyrethroids are voltage-gated sodium channels (Na_V_s, or *para*). Na_V_s are made of four transmembrane domains (DI to DIV), each of which contains six transmembrane helices (S1 to S6) connected with intracellular and extracellular loops. S4 helices in the voltage sensor domain (S1–S4) are sensitive to depolarization due to the presence of positively charged residues arginine or lysine every three amino acids and P-loops between helices S5 and S6 form the pore domain of the channel^18,19^. Identification of point mutations associated with resistance to pyrethroids and approaches of computer modelling allowed localization of putative pyrethroids binding sites involving two cavities located in the transmembrane domains of the Na_V_s. The first cavity includes S4-S5 linker and helix S5 from domain I and S6 helix from domain II while the second cavity comprises residues from S4–S5 linker, the helix S5 and possibly the P-loop of domain II, and the helix S6 from domain III^19–26^ (Fig. 1A). This binding perturbs the normal functioning of the Na_V_ channel and causes the development of a slow deactivating tail current that constitutes the functional signature of pyrethroids.

Recent heterologous expression studies on Na_V_s suggest a higher sensitivity of honey bees as compared with bumble bees, *Varroa destructor* and cockroaches channels to one widely used pyrethroid, tau-fluvalinate^27–29^. However, the question remains as to whether the differential sensitivity seen using the *Xenopus* oocyte expression system can be confirmed in native neurons. We compared the biophysical properties of the sodium channels from the antennal lobe neurons (ALNs) of *Am* and *Bt* and analysed the functional effects of two pyrethroids, tetramethrin and esfenvalerate. Biophysical analysis and numerical simulations suggest that pyrethroids cause changes in the transition rates between channel functional states, but with marked specificities between these two bee species.

## Results

### Na_V_s of *Apis mellifera* and *Bombus terrestris* share similar biophysical properties

**Figure 2:**
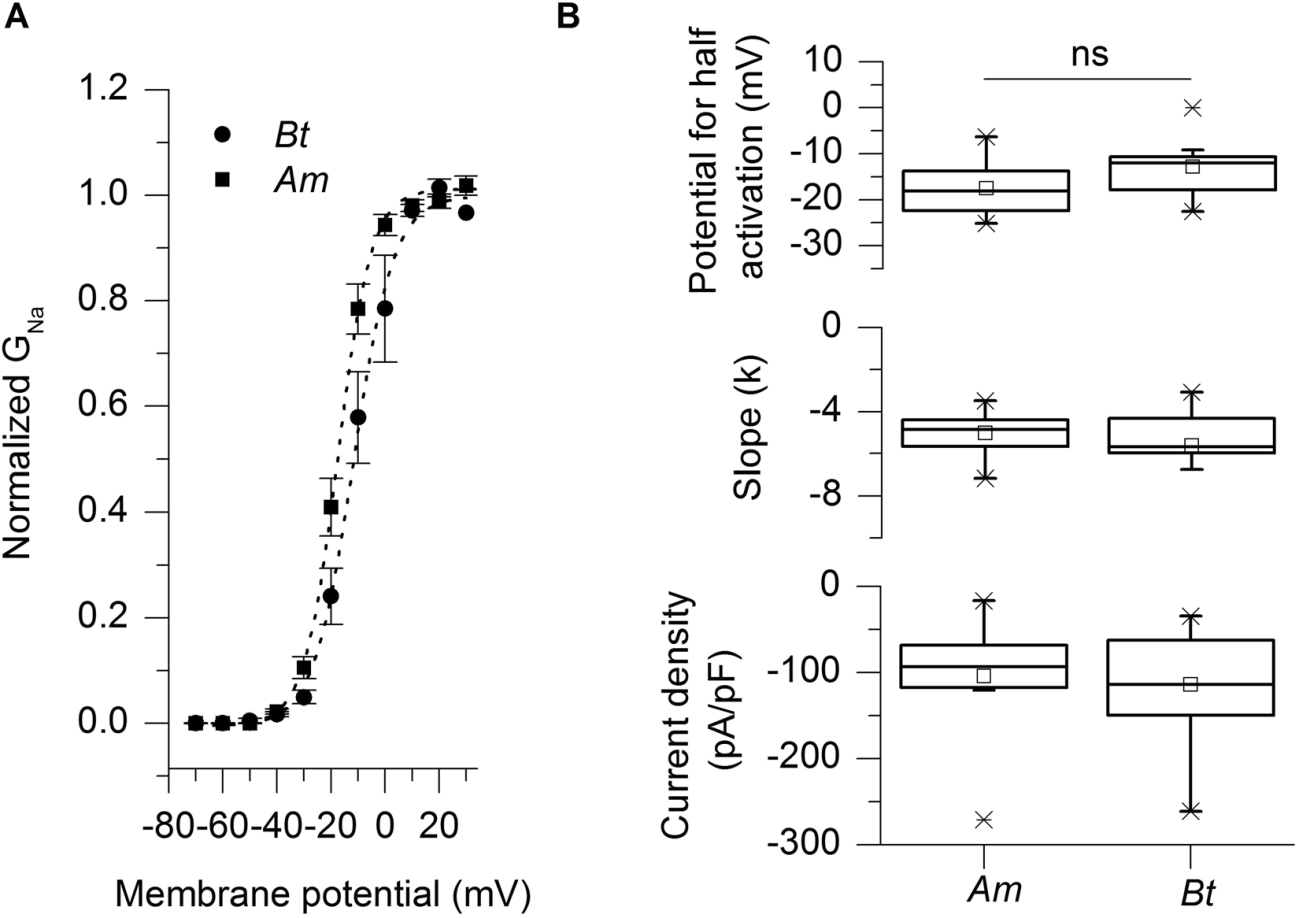
Biophysical properties of voltage-gated sodium channels from *Apis mellifera* and *Bombus terrestris*. (**A**) Mean steady state activation curves for *Am* (squares) and *Bt* (circles) fitted with the Boltzmann equation. This generated the parameters shown in panel (B). No significant difference was detected between *Am* and *Bt*.

Voltage-gated sodium channels of *Am* (NCBI GenBank Accession AMB38675.1) and those from *Bt* (NCBI Reference Sequence: XP_012167116.1) share 97.3% homology (Genestream Search network server, IGH Montpellier, France^30^ and multiple amino acid sequences alignment show a remarkable conservation for the putative pyrethroids binding sites across bee species (Fig. 1B). A few differences in amino acids sequences are seen in the intracellular loops between domains I-II and III-IV and S4 from domain III (magenta, Fig. 1C), but these amino acids residues are not directly involved in ligand binding on the putative sites^19^. Supplementary Table 1 recapitulates amino acid differences between the two sequences. We have investigated some biophysical parameters of the Na_V_s in *Am* and *Bt* (Fig. 2). On average, the maximal sodium current densities were similar in both species with −104 ± 16 pA/pF (n = 16) and −114 ± 19 pA/pF (n = 12) for *Am* and *Bt*, respectively (Mann-Whitney test, p = 0.6195). The potential for half activation (Vm) was −17.4 ± 1.4 mV (n = 19) for *Am* and was not significantly different from the value obtained for *Bt* (−12.8 ± 1.9 mV, n = 13; Mann- Whitney, p = 0.1223). The slope factors (k) of the activation curves were not statistically different either: 5.1 ± 0.2 (n = 19) for *Am* and 5.6 ± 0.6 (n = 13; p = 0.4331) for *Bt*.

The time constant of fast inactivation, which was measured at the maximal amplitude of the sodium current, was not different either across species: 0.37 ± 0.04 (n = 9) and 0.32 ± 0.03 ms (n = 8) for *Am and Bt*, respectively (t-test, p = 0.2872). Therefore, despite the presence of some differences between the two species, and in particular the addition of a positive charge at the bottom of DIII-S4, we did not observe any significant difference between the two species on these parameters.

### Tetramethrin modifies Na_V_s in *Am* and *Bt* antennal lobes neurons

**Figure 3:**
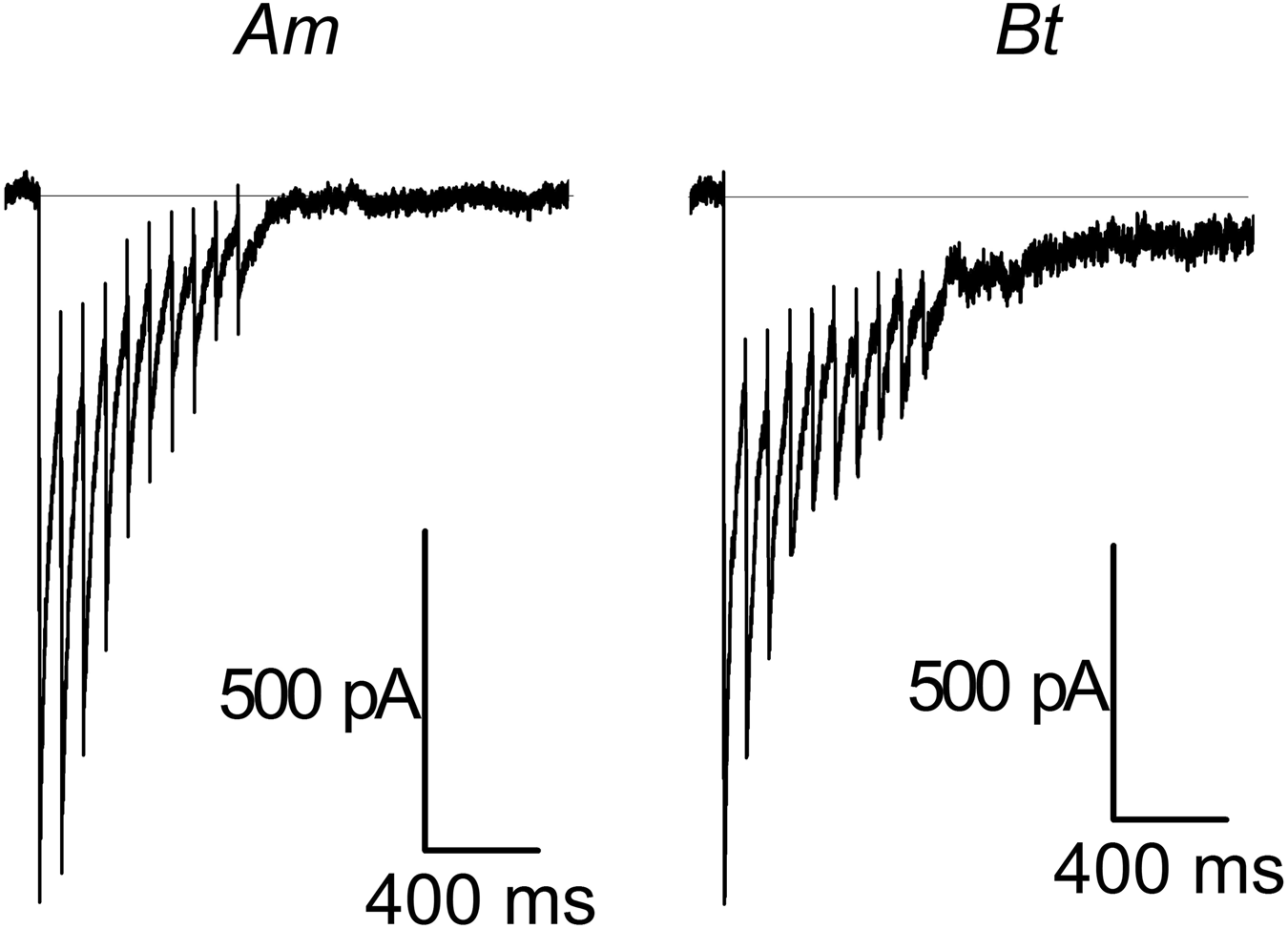
Tetramethrin induces a tail current in ALNs from *Bt* and *Am*. Sodium current recorded in ALNs in response to a train of ten short depolarizations (3 ms) in *Am* (left) and *Bt* (right) after perfusion of tetramethrin 10 µM. The tail current is the hallmark of the interaction of pyrethroids with voltage-gated sodium channels.

Following repetitive short (3 ms, 13 Hz) depolarizations, tetramethrin at concentration 10 µM induced a tail current in both *Am* and *Bt* ALNs (Fig. 3). For both species, tetramethrin reached its maximal effect within the first to third depolarizations, then the tail current’s amplitude progressively decreased. However, after the train of depolarization, the tail current was more sustained in *Bt*.

**Figure 4:**
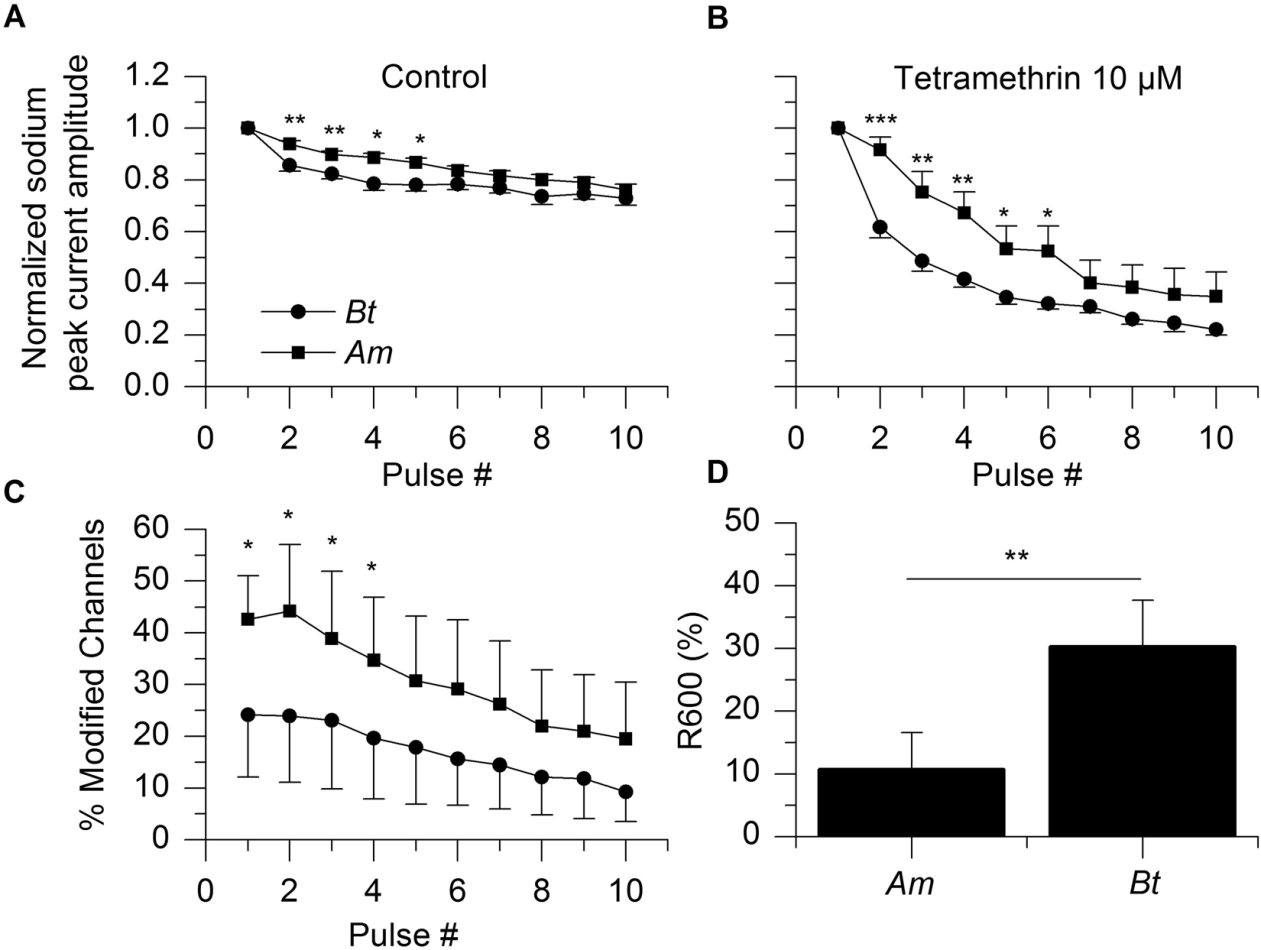
Sensitivity of ALNs from *Bombus terrestris* and *Apis mellifera* to tetramethrin. (**A**,**B**) Use-dependent decrease in the sodium peak current amplitude in control condition (left) and with tetramethrin 10 µM (right; *p < 0.05; **p < 0.01; ***p < 0.005). Scales on Y axis are identical. (**C**) Mean percentages of modified voltage-gated sodium channels in tetramethrin-exposed ALNs (*p < 0.05). (**D**) Decay of the tetramethrin-induced tail current after ten consecutive depolarizations. The decay of the tail current is represented by the parameter R600 which is the percentage of the tail current that remains 600 ms after the end of the tenth depolarization. Tetramethrin-induced tail current decayed faster with *Am* ALNs than with their *Bt* counterparts (**p < 0.005).

We have examined the sodium peak amplitude during depolarization pulses in the presence of tetramethrin. Over the course of a single short depolarization, tetramethrin caused a decrease in the sodium peak current in *Am* as compared with control: −27 ± 12% (n = 10; p = 0.0175; paired t-test). It decreased the sodium peak current amplitude in *Bt* ALNs as well, but this decrease was not statistically significant: −25 ± 31% (n = 8; p = 0.1057). Upon multiple depolarizations, the sodium peak current amplitude recorded within the pulse decreased in control conditions (Fig. 4). An interspecific comparison showed that the sodium current from *Bt* decreased slightly, but significantly faster than the one from *Am* during the first four depolarizations. Still, 80% of the sodium current remained in both species after ten consecutive depolarizations (t-test, p = 0.17; Fig. 4A). Tetramethrin significantly accelerated the decrease process for both species, the remaining sodium current after ten consecutive depolarizations is not significantly different: 35 ± 9% (n = 10) and 22 ± 2% (n = 8) for *Am* and *Bt*, respectively (Mann- Whitney, p = 0.5573; Fig. 4B).

If we consider the percentage of modified channels, *Am* is significantly more vulnerable to tetramethrin than *Bt*, but only if the comparison is restricted to the first four depolarizations (Fig. 4C). The percentages of modified channels were 42.6 ± 8.5 (n = 10) and 24.2 ± 12.1 (n = 8) at the first depolarization for *Am* and *Bt*, respectively (Mann- Whitney p = 0.025). At the tenth depolarization, the percentages of modified channels were at the same level: 19.5 ± 10.9 (n = 10) and 9.2 ± 5.7 (n = 8) for *Am* and *Bt*, respectively (p = 0.3949).

Other parameters such as the decay of the tail current may also influence the outcome of an exposure to a pyrethroid. The decay of the tail current is one of the canonical parameters used to characterize the effects of pyrethroids. Here, that decay was estimated by measuring the remainder (in %) of the tail current 600 ms after the end of the tenth depolarization (R600; Fig. 4D). Tetramethrin-induced tail current decayed significantly faster in *Am* than in *Bt*, R600 for tetramethrin being 10.8 ± 5.8% (n = 10) for *Am* and 30.3 ± 7.5% (n = 8) for *Bt* (Mann- Whitney test, p = 0.0027).

The development of a tail current that decays slowly in the presence of pyrethroids produces a higher amount of sodium charges entering the neurons’ cytoplasm as compared with control conditions. The total amount of sodium charges (Qtot) is another way of assessing current modification during neuronal activity. Upon a train of ten successive depolarizations at a 13 Hz frequency, Qtot was calculated in the presence of tetramethrin (Supplementary Figure 1). The Qtot in presence of tetramethrin was significantly increased by 5.6 and 7.1 fold as compared with Qtot in control conditions for *Am* (paired t-test, p < 0.0001) and *Bt* (p < 0.005), respectively, but no species-specific difference was observed regarding the amount of charges in the presence of tetramethrin 0.14 ± 0.02 nC (n = 10) and 0.20 ± 0.06 nC (n = 8) in *Am* and *Bt* neurons, respectively (t-test, p = 0.3382).

**Figure 5:**
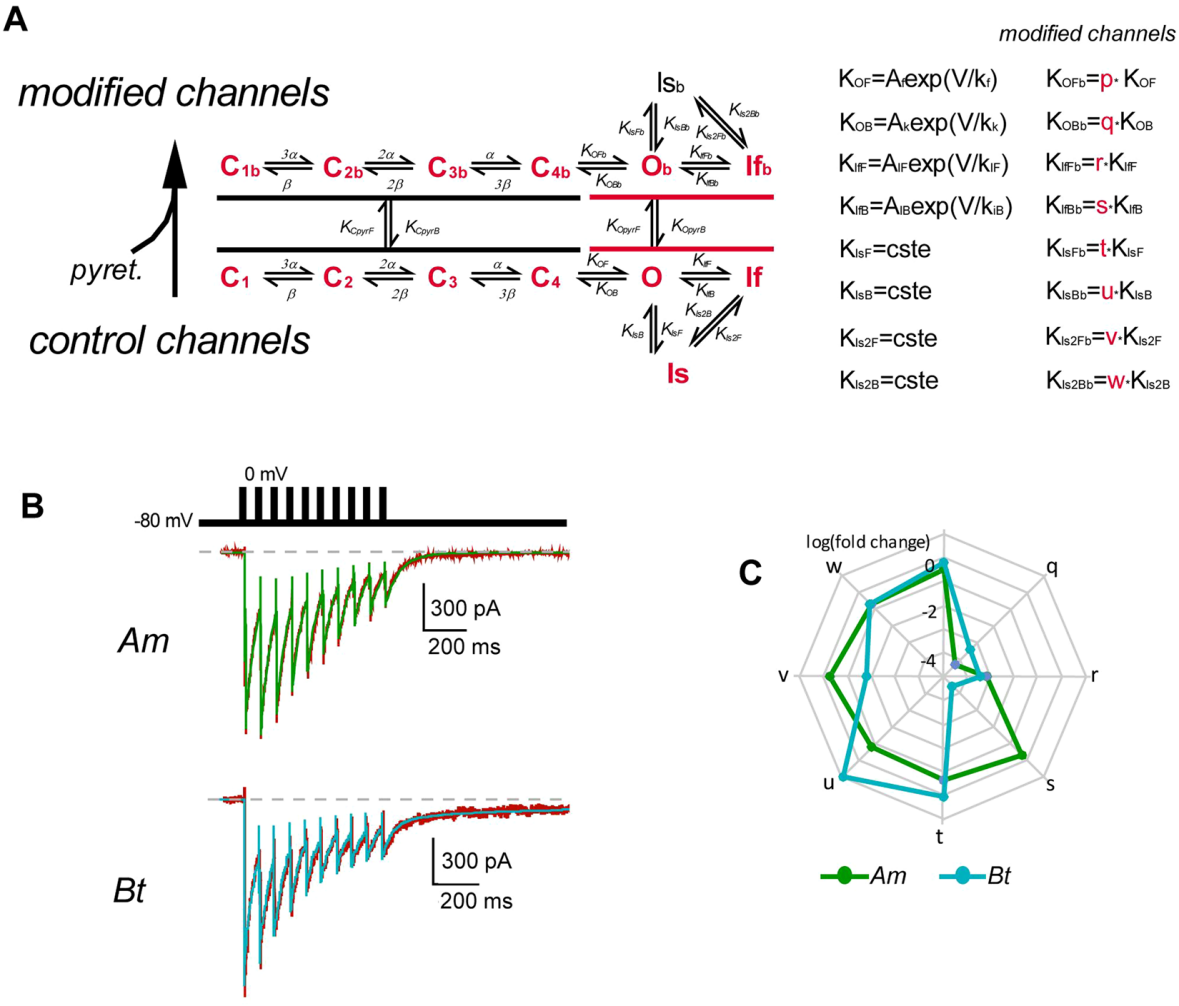
Numerical simulation of Na_V_s states transitions in the presence of tetramethrin. (**A**) State model used to fit the experimental traces. Pyrethroids can bind to either a closed (black) channel or an open one (red). Once bound they modify channel kinetics to and from open and inactivated states by factors p, q, r, s, t, u, v and w, respectively, to give K_OFb_, K_OBb_, K_ifFb_, K_ifBb_, K_isFb_, K_isBb_, K_is2Fb_ and K_is2Bb_. (**B**) Traces (green and blue) obtained from model above are superimposed to representative experimental traces (red) in the presence of tetramethrin. This allows determining the changes in the different kinetic parameters introduced in panel (A). (**C**) Radargraph of the changes (logarithmic scale) in the different kinetic parameters produced by tetramethrin in *Am* (blue line) and *Bt* (orange line).

At this point, our experimental data have shown significant differences in the action of tetramethrin on the Na_V_s of both bee species, but the interpretation of the species-related differences in the toxicity of these molecules appears quite challenging. Indeed, with respect to the percentage of modified channels, tetramethrin tended to be more effective in *Am* than in *Bt* Na_V_ channels, but if the decay of the tail current is considered, the slower-decaying tetramethrin-induced tail current in *Bt* neurons may have more deleterious effects in that bee species than in *Am*. Therefore, these two parameters do not provide a clear-cut answer. We took advantage of the Markovian model to gain further insight into the kinetics changes of pyrethroid-bound sodium channels in both bee species (Fig. 5A).

### Kinetics of modified voltage-gated sodium channels

Fit of the Markovian model to experimental recordings in control conditions gave a set of values that allow to mimic the kinetics of sodium current traces as well as activation and inactivation curves and behaviour of the channel during a train of depolarizations. As expected from the similarity in the biophysical properties of the two Na_V_ channels, most of the kinetics parameters governing the transition rates between the seven different functional states (C1-4, O, If and Is) were similar. The only exceptions were transition rates to slow inactivated (K_IsF_) and from the fast and slow inactivated state (K_Aib,_ K_IsB_) with smaller values for *Bt* (Supplementary Figure 2).

In the presence of tetramethrin, adjustment of the model was made on a representative experimental recording in terms of sodium peak amplitude and its inactivation, tail current amplitude, decay of the tail current (Fig. 5B). Changes in the transition rates resulting from the binding of tetramethrin are given in Fig. 5C (factors p, q, r, s, t, u, v and w, see Methods). Briefly, of the 8 parameters, only 3 were clearly differentially affected (>2 log in differences) in *Am* and *Bt*. Deactivation of the voltage-gated sodium channels, mainly directed by K_oB_ was affected in both species, giving rise to this slow-deactivating tail current, but *Am* was more sensitive by a factor ~7 (q values of 17,000 and 2,200 for *Am* and *Bt*, respectively). Important changes in the forward and backward rates to fast inactivation (transition from O to If) were also recorded: the forward transition (O to If, r) was decreased by factors 1,300 and 2,500 for *Am* and *Bt*, (r = 7 10^−4^ and 5 10^−4^), respectively, with no major difference between the two species; the reverse transition (If to O, s) was almost unaffected in *Am* (a decrease by a factor 2, i.e. s = 0.5), but markedly impacted by a factor greater than 25000 (s = 3.8 10^−5^) in *Bt*. The slow pore-dependent inactivation (Is) can develop either from the Open state (O, Slow inactivation Mode 1) or the fast inactivated state (If_,_ Slow inactivation Mode 2). The changes in the forward and backward transition rates to Slow inactivation Mode 1 produced by tetramethrin (factors t and u, respectively) were of minor amplitude, and not dramatically different between the two species (t = 0.2 and 1.13, u = 0.15 and 8.8 for *Am* and *Bt*, respectively). For the Slow inactivation Mode 2 (If to Is), changes in the forward transitions v were 0.5 and 1.7 10^−2^) for *Am* and *Bt*, respectively, while the backward transition was hardly affected (w = 0.2) and similar for the two species.

**Figure 6:**
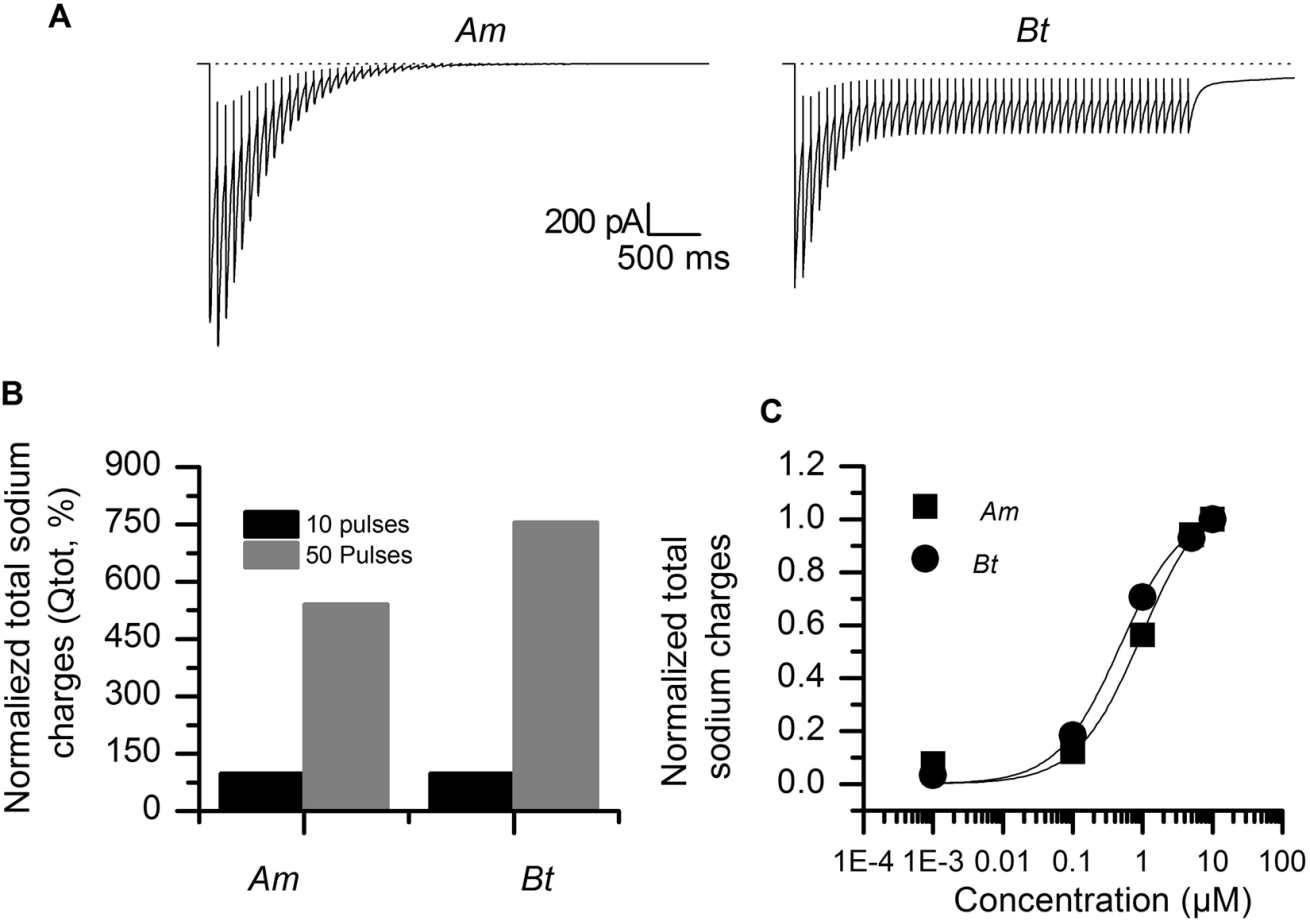
Effect of an increase in neuronal activity in the presence of tetramethrin. The sodium current was computer-generated in the presence of tetramethrin, using the Markovian model. (**A**) Computer-generated sodium current in the presence of tetramethrin and in response to 50 consecutive depolarizations at 13 Hz. (**B**) The total sodium charges (Qtot) increased with 50 pulses (grey) as compared to 10 pulses (black). (**C**) Concentration dependence of the total sodium charges in *Bt* (filled circles) and *Am* (filled squares) for 50 depolarizations at 13 Hz.

The use-dependent action of pyrethroids (see Fig. 4) suggests that the level of neuronal activity greatly influences the outcome of an exposure to pyrethroids. Furthermore, since, we did not see any difference between the two bee species in terms of the total amount of sodium charges (Qtot, see Supplementary Figure 1) we wondered whether the level of neuronal activity can differentially influence that parameter in the two species. Using the Markovian model, we simulated an increase in the neuronal activity by increasing the number of depolarizing pulses to 50 while keeping the frequency at 13 Hz. This exacerbated the differences between *Am* and *Bt* that were first seen during the smaller train of depolarizations (Fig. 6A). With tetramethrin, the amplitude of the tail current decreased and was nullified rapidly in *Am* whereas in *Bt*, the amplitude of the tail current gradually decreased upon the first ten consecutive depolarizations then it reached a plateau. As with our experimental data (see Fig. 3), the tail current generated with the Markovian model decayed much slower in *Bt* than in *Am*. We also noticed a large increase in the total sodium charges (Qtot, Fig. 6B). As compared to 10 pulses, Qtot in the presence of tetramethrin was increased by factors 5 and 7.5 in *Am* and *Bt*, respectively, when we increased the number of depolarizations to 50. All the above does suggest two different modes of action, with a rapid inhibition of functional channels in the case of *Am*, while the channels in *Bt* are still active. In the latter case, sodium channels will still end-up completely inactivated in non-voltage-clamp conditions due to massive entry of sodium into neurons, and the consecutive strong depolarization of the neuron. Using extracted Kcpyr and Kopyr dissociation constants, we simulated an increase in ligand concentration and constructed a concentration-dependent increase in Qtot in both bee species. The EC50 values obtained from these curves were 0.97 µM for *Am* and 0.47 µM for *Bt* (Fig. 6C). The small leftward shift of the curve and the higher Qtot values in *Bt* suggest a greater sensitivity of the bumblebee sodium channels to tetramethrin than those from honey bee.

**Figure 7:**
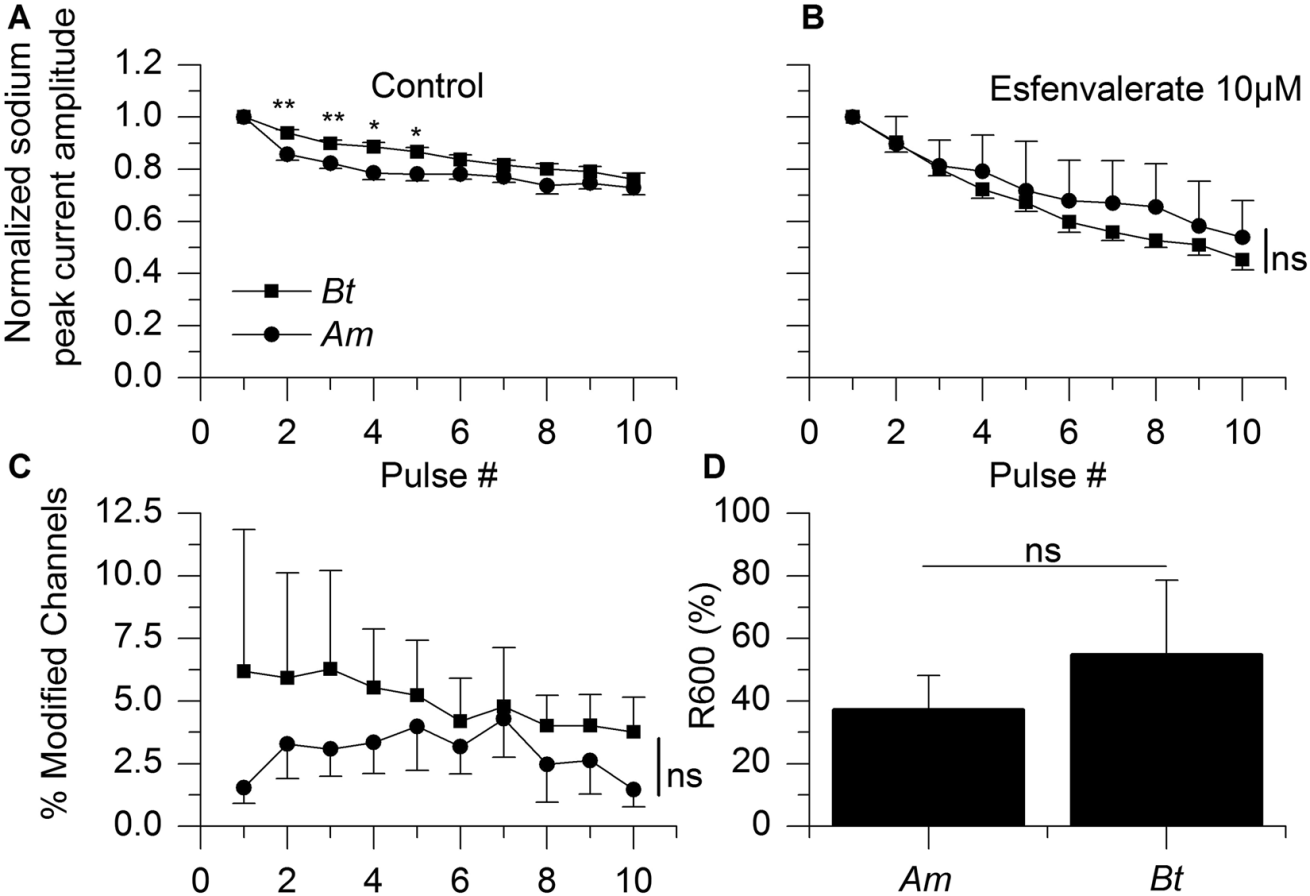
Sensitivity of ALNs from *Bombus terrestris* and *Apis mellifera* to esfenvalerate. (**A**,**B**) Use-dependant decrease in the sodium peak current amplitude in control condition (left; (*p < 0.05; **p < 0.01) and with esfenvalerate 10 µM (right). Scales on Y axis are identical. (**C**) Mean percentages of modified voltage-gated sodium channels in esfenvalerate-exposed ALNs. (**D**) Decay of the pyrethroid-induced tail current after ten consecutive depolarizations expressed as R600. No species-dependent difference was detected. ns = not significant.

Tetramethrin being one of the archetypical molecule used for the study of the effects of pyrethroids, we examined whether its effects on sodium channels would match those of another pyrethroid, esfenvalerate in both bee species. Esfenvalerate is a type II pyrethroid used in orchards for protection against beetles and lepidopterans among others. In the presence of esfenvalerate, the sodium peak current amplitude decreased after one depolarization by 44 ± 8% (Wilcoxon match paired rank test, n = 8; p = 0.0078) in *Am* and by 28 ± 24% (n = 3; p = 0.5) in *Bt*. This decrease was amplified by repeated depolarizations (13 Hz) in *Am* (Mann- Whitney, p = 0.0011) and no species-wise difference was observed since the remaining sodium current at the tenth depolarization was 45 ± 4% (n = 8) and 54 ± 14% (n = 3) for *Am* and *Bt*, respectively (p = 0.6303; Fig. 7B). We then calculated the percentage modified channels in esfenvalerate-exposed neurons. Again, we did not detect any significant species-wise difference (Fig. 7C). In both species, esfenvalerate modified significantly less voltage-gated sodium channels than tetramethrin and the difference between tetramethrin and esfenvalerate was more pronounced with *Am* (see Supplementary Figure 3). As to the decay of the esfenvalerate-induced tail current (expressed as R600), no significant species-related difference was seen (Mann- Whitney, p = 0.424, Fig. 7D). Therefore, as opposed to tetramethrin, esfenvalerate did not produce any species-specific effects.

## Discussion

This is the first comparative study of the biophysical and pharmacological properties of the voltage-gated sodium channels from antennal lobe neurons of two bee species, *Am* and *Bt*. The homology in the sodium channels amino acids sequences between the two bee species is 98%, with most of the differences restricted to the intracellular loops between domains I-II and III-IV and S4 from domain III (Fig. 1 and Supplementary Table 1). These locations may not be critical for channel permeability, but could be involved in some aspects of the channel gating. However, sodium channels from *Am* and *Bt* have very similar biophysical properties under control conditions, suggesting that in the absence of pyrethroids, the differences in amino acids sequences do not impact the channel activity in a significant way.We compared the cellular efficiency of representative pyrethroids on *Am* and *Bt* to explore their differential effects on bee species. For this purpose, esfenvalerate was chosen because it is currently used in agriculture (including in the EU) so that *Apis* and *Bombus* have a similar risk to be exposed to it. It possesses a phenoxyphenyl radical just like 85% of pyrethroids authorized in Europe (this radical is involved in the molecular interaction with sodium channels) and it has a unique chlorobenzyle radical (a property it shares with no other pyrethroid, except the tau-fluvalinate molecule). In addition, it is a type II pyrethroid since it has a cyanide radical (-CN), and it induces specific type II toxicological symptoms in insects. Importantly, its persistence in the field is intermediate (DT50 of 19 days, as compared with 3.5 days for tau-fluvalinate according to the PPDB database^31^) and its toxicity for bees is quite high (LD50 equals 0.06 µg/bee). The other molecule we have selected – tetramethrin - is a type I pyrethroid (no cyanide radical) inducing type I toxicological symptoms and it possesses the dimethylcyclopropane radical, an important toxophore in 70% of authorized pyrethroids, involved in the molecular interaction with sodium channels. Whilst tetramethrin has been withdrawn (not approved anymore for field use in the EU, but may be used elsewhere around the world), i) it is probably one of the most canonical pyrethroids initially used to characterize the mode of action of this insecticide class on neurons (works from the group of Narahashi) and ii) it is still widely used in household aerosol insecticides, as it is very efficient and with a rapid action on wasps and hornets (instantaneous knockdown), iii) its toxicity for honey bees is pretty high^9^.

During pyrethroids perfusion, channels from both species were affected in their slow inactivation and deactivation kinetics with a pronounced slowing of these two parameters. Channels from both species were also more sensitive to tetramethrin (a type I pyrethroid) than to esfenvalerate (a type II pyrethroid) with regard to the percentage of modified channels. Conversely, the esfenvalerate-induced tail current decayed slower than the tetramethrin-induced tail current, which is consistent with the properties of type I pyrethroids, known to induce a faster decaying tail current than type II pyrethroids^32,33^. Besides these qualitative similarities in between the two species, experimental recordings revealed however profound interspecific differences in the behaviour of the channels of these two species when challenged with tetramethrin. Indeed, the percentage of modified channels by tetramethrin was higher in *Am* neurons than in *Bt* neurons, but on the contrary, the slowing of the deactivation tail current was more pronounced in *Bt*. In *Xenopus* oocytes, the honey bee sodium channels are differentially sensitive to fenvalerate and permethrin^34^, which concurs with our present results with tetramethrin and esfenvalerate. In *Xenopus* oocytes, 10 µM tau-fluvalinate modified 50% of AmNa_V_ channels^28^, and ~55% of *Bombus impatiens* channels^29^ after a 100-pulses protocol. This suggests that *Bombus impatiens* and *Apis mellifera* are equally susceptible to tau-fluvalinate. Interestingly, the group of Dong showed evidence that *Bombus impatiens* Na_V_s were less sensitive to etofenprox (1 µM), another synthetic pyrethroid, than Na_V_s from cockroach, fruit fly and mosquito^29^, a result which concurs with our results showing differential effects of tetramethrin on *Am* and *Bt* neurons.

The computer modelling allowed us to confirm our experimental data and detect differences in the kinetics parameters of the sodium channel that were harder to notice using conventional analysis. We were able to evaluate both the changes in the kinetics parameters produced by the drug and the differences in the response between the two bee species. Transitions rates from the Open state were the most affected in the two bee species with striking differences in the kinetics parameters governing the deactivation (O to C, q), the slow inactivation Mode 2 (If to Is, v) and the recovery from fast inactivation (If to O, s). In addition to the deactivation, the most outstanding difference between the two species is undoubtedly the recovery from fast inactivation, which is almost not affected in *Am*, but decreased by 25,000 in *Bt* (Fig. 5). The functional effect of the drugs over longer stimulations was clearly different in the two species. In *Am*, the cumulative decrease of the amplitude of the tail current probably due to slow inactivation (see Fig. 4B for instance) and the rapid decay of that tail current led to a rapid extinction of channel activity. In *Bt*, despite the slow inactivation of the sodium current, the plateau-like decay of the tail current allows sodium channels to remain active during the period of the stimulation. In physiological situation, the larger amount of sodium ions entering the neurons in *Bt* may produce a stronger membrane depolarization, and thus a rapid inactivation of the sodium channels. Therefore, albeit the functional effect at the channel level *per se* appears quite different between the two species one can speculate that the final result in both cases will be a neuronal depolarization ending up with sodium channel inactivation and blockade of action potential propagation. The amino acids sequences of the sodium channels from the two species are perfectly conserved in the putative pyrethroids binding sites (Fig. 1 and^29^), but the non-conservation of some amino acids residues in helices S2, S3 and S4 of the domain III between *Am* and *Bt* sodium channels (see Supplementary Table 1) may in part explain these functional differences. These data suggest that the structure of the pyrethroids binding site within the channel pore is probably not the only factor modulating the effect of the drugs. Amino acid variations in regions distant from the putative pyrethroids binding site may for instance decrease the accession of the molecule to its binding site within the channel, induce changes in some gating parameters affecting the efficiency, but not the affinity of the drug, or affect, *via* long-distance allosteric mechanisms the binding site.

Although the percentage of modified channels is the traditional way of estimating the effects of pyrethroids on voltage-gated sodium channels, it is mainly related to channel affinity for pyrethroids and other factors can affect the relative toxicity of pyrethroids from one species to another. As we have seen, the decay of the tail current can greatly influence the functionality of voltage-gated sodium channels and eventually the amount of sodium charges (Qtot) that enters the neuron’s cytoplasm. Qtot is a composite parameter that takes both the percentage of modified channels (i.e. channel affinity for the drug) and the speed of the decay of the tail -current (drug efficiency) into account; therefore, Qtot may be a more appropriate parameter to estimate the effects of pyrethroids on neurons. Our results suggest that *Bt* is more susceptible to pyrethroids than *Am*. Classical toxicological data with the pyrethroid λ-cyhalothrin support this result, but not with other members of the pyrethroid class such as deltamethrin and permethrin^35^. When considering available LD50 for all wild bee species (including *Bt*), it turns out that the toxicological profile is not clear for several pyrethroids. For instance, whereas α-cypermethrin, cyfluthrin and deltamethrin are consistently more toxic for *Am*, other pyrethroids such as cyhalothrin, λ-cyhalothrin and permethrin are either more or less toxic for *Am*, revealing the difficulty of establishing general toxicological rules between *Apis mellifera* and other bee species^35^. Yet it should be noted that the mortality rate is not necessarily the only relevant parameter to assess long term risks associated with pyrethroids since bees can also be exposed to sublethal doses of insecticides^36,37^. Therefore, that parameter does not reflect the subtleties in the action of insecticides when bees are exposed to small doses of these compounds. The sublethal effects of pyrethroids on *Am* have been extensively investigated and they include impairment in memory and learning performances^6,7^, disorientation^8^ and loss in locomotor capabilities^9^. This can possibly lead to subtle, but persistent adverse consequences on the colony since insecticides can interfere with facets of the colony properties such as the larval development, the feeding behaviour or olfactory discrimination^38^, for review, see^39^. Regarding the bumblebees, sublethal data are rather scarce, but some behavioural aspects of the action of pyrethroids have been investigated. In an experiment in which the foraging activity of bumble bees was monitored using RFID tracking, individuals exposed to pyrethroid lambda-cyhalothrin carried out longer foraging expeditions than unexposed individuals^16^. This could be the sign of impairments in the bumblebee’s memory, orientation and/or flight performances. More behavioural studies need to be carried out to root out eventual differences in sensitivity of *Am* and *Bt* at sublethal level. Other factors for instance, the ability of pyrethroids to get through the cuticle barrier or the metabolism of pyrethroids may also influence the sensitivity of the bees to these compounds. Life history traits of bee species and their level of sociality may impact their vulnerability as well, with *Apis mellifera* being a complex eusocial species (like *Apis dorsata*, *Apis florea* and *Melipona quadrifasciata*), *Bombus terrestris* showing an obligate simple eusociality and other species being solitary species such as *Habropoda laboriosa* or *Megachile rotundata*. The life cycles of *Apis mellifera* and *Bombus terrestris* differ in numerous aspects: unlike domestic honey bees, bumblebee queens live part of the year outside of a colony and exposure to pesticides may have a greater impact on bumblebees’ colony fitness as compared with that of honey bees. For a period in their life cycle, bumblebees lack the “buffer” that domestic honey bees benefit from in their colony^40,41^.

In conclusion, using *in vitro* and *in silico* approaches provide essential clues as to the differences in sensitivity between the domesticated honey bee *Am*, the bumblebee *Bt* and possibly other bee species to pyrethroid insecticides. *Am* cannot necessarily be considered as a proxy for other bee species and the effect of one particular pyrethroid cannot be extrapolated to those of other members of the family. These differences in species and molecules sensitivity should be taken into account in the implementation of regulatory tests if they are to better inform environmental risk assessors.

## Material and Methods

### Cell culture

Honey bees colonies were raised in the Department apiary in Avignon and bumblebee colonies were purchased from Koppert (www.koppert.fr). The bees were picked up at the pupal age, 4-6 days before adult emergence. For sterility purpose, pupae (5 to 7 days old) were briefly rinsed with alcohol (70%) and sterile water. Antennal lobes were isolated from brains in a Ca^2+^- Mg^2+^-free solution (see Solutions). A hyperosmotic non-enzymatic dissociation in calcium and magnesium-free physiological solution (500 mOsm/l, 4 °C; 15 min) and a centrifugation (1800 rpm, 22 °C, for 3 min) followed. Antennal lobes fragments were placed in the culture medium described below and triturated with a p100 pipette. Neurons were plated on poly-L-lysine coated Petri dishes, and cultured following the hanging drop method (29 °C, high humidity)^12^. Experiments were performed at room temperature (20–22 °C) at 2–5 days *in vitro*.

### Electrophysiological recordings

The sodium currents were measured in the whole-cell configuration, with a patch-clamp amplifier (RK400, Bio-Logic, Claix, France) operated with WinWCP (John Dempster, Strathclyde University, UK) and an analog/digital board (PCI-6014, National Instruments, Austin, TX, USA). Pipettes were made from borosilicate capillaries with a puller (P30, Sutter Instruments Co, Novato, AS, USA). Sylgard was added at the pipette tip to minimize its capacitance. Electrodes filled with the intracellular solution had a resistance of 5–7 MΩ. Junction potential was compensated before seal formation and the residual microelectrode capacitance was compensated for. Cell capacitance was nulled and series resistance was maximally compensated for (~60–80%).

Trains of successive depolarizations (test pulses) at 13 Hz consisted in short stimulations (3 ms; from a resting potential of −80 mV to −10 or 0 mV) with an inter-pulse (duration between the initiation of two successive pulses) of 78 ms. Passive leak currents and residual linear capacitive currents were subtracted using a P/4 protocol where each test pulse was preceded by a series of 10 pulses (with same duration and interpulse as the test pulses) which amplitudes were one fourth in the opposite direction. Patch-clamp data were analyzed with OriginPro software.

Steady-state activation data were fitted with the Boltzmann equation: m = 1/(1 + exp[(V-Vm)/k]) where V is the potential to which the membrane is depolarized, Vm is the potential for half activation and k is the slope parameter. The percentage of voltage-gated sodium channels modified by pyrethroids was calculated using the equation: M = [(Itail/(Eh-ENa))/(INa/(Et-ENa))]x100 where M is the percentage of modified channels, Itail is the maximal tail current amplitude (measured 3 ms after the end of the test pulse with an Origin custom script, OriginLab corp.), Eh is the potential to which the membrane is repolarized, INa is the amplitude of the sodium current measured in control conditions during the test pulse, Et the membrane potential during the test pulse and ENa the calculated equilibrium potential of the sodium ion^42^.

Statistical analyses were performed with GraphPad Prism (version 6 for Windows, GraphPad Software, La Jolla California USA; http://www.graphpad.com). The Student t-test was used when data were normally-distributed, otherwise, the Mann- Whitney or the Wilcoxon tests were used. We considered statistical significance for p < 0.05. Averaged data are given as mean ± s.e.m.

### Solutions

A calcium and magnesium-free physiological solution was used for dissection and it contained (in mM): 140 NaCl, 5 KCl, 10 HEPES, 90 Sucrose (pH 7.2, 400 mOsm/l). The hyperosmotic physiological solution for neurons dissociation was 500 mOsm (adjusted with sucrose). The culture medium was made of the commercially-available Leibovitz’s L-15 medium (Thermo Fisher Scientifics) supplemented with 5.5 mM D-Glucose, 3.3 mM L-proline, 75 mM sucrose, 10% FBS, 1% penicillin/streptomycin (pH7.2, 400 mOsm/l). Sodium currents were recorded in an extracellular solution containing (in mM): 120 NaCl, 20 TEA-Cl, 2 MgCl_2_, 2 BaCl_2_, 0.1 CdCl_2_, 1 4-aminopyridine, 10 HEPES, 90 Sucrose (pH 7.2, 400 mOsm/l). In all experiments with pyrethroids, the control extracellular solution contained DMSO (0.1%). The pipette solution contained (in mM): 135 CsCl, 5 NaCl, 1 MgCl_2_, 1 CaCl_2_, 10 EGTA, 10 HEPES, 90 Sucrose (pH 7.2, adjusted with CsOH, 380 mOsm/l). We used a glass tubing inspired by procedures from Tatebayashi and Narahashi^42^ to superfuse neurons with pyrethroids. Perfusion rate was approximately 1 ml/min. Only one neuron was recorded per dish and then the tubing system was discarded after each series of perfusion and the reference electrode were abundantly rinsed with running 70% ethanol and distilled water. Tetramethrin (CAS number: 7696-12-0) and esfenvalerate (CAS number: 66230-04-4) were purchased from Sigma-Aldrich Co (St-Louis, MO, USA). Stock solutions (10 mM) were prepared in DMSO (vortexed and briefly sonicated to fully dissolve the compounds) and dissolved in the perfusion solution. Drugs were applied 1 min prior the beginning of the train of depolarizations (3 ms long at 13 Hz as above).

### Markovian model

The Markovian model used in this work is essentially the same as previously described^12^ (Fig. 5A, left). The 3 voltage-dependent activations (forward α and backward β) of the three similar S4 segments necessary for the channel to open, connect the 4 Closed states (C), with a final transition, also voltage-dependent (with forward K_oF_, and backward K_oB_ rates) to the open state (O)^43^. Voltage-dependent fast inactivation (due to the loop between III and IV domains) relies in the activation of the fourth S4 (DIVS4, with transition rates K_ifF_, K_ifB_^44^). Non voltage-dependent transition rates to and from the slow, pore-dependent inactivated state (Is) are K_isF_ and K_isB_ for transition to (O), and K_is2F_ and K_is2B_ for transition to (If), respectively. Voltage-dependent transition rates are set as A.exp(V/k), where A is the value at V = 0, k is the voltage dependency, and V the membrane potential. The fit to the experimental data recorded in control conditions with this set of differential equations, gives a set of values able to reproduce most of the channel properties. Pyrethroids binding can occur on the Open state, as suggested from their use-dependent effects and the location of the putative pyrethroids binding site within the channel pore^22,24^. The possibility of the pyrethroids binding to the Closed states also exists^32,45^. These two possibilities are introduced in our study.

We limit the change in the kinetic parameters affected by pyrethroid binding to the channel, to those affecting the pore module where the putative binding site is located (S4-S5 linkers of the DI and DII, and S5 of DI and DII, and S6 of DII and DIII)^22,24^ *i.e*. transition rates to and from Open (O) and Inactivated (If and Is) states. Only changes in amplitude are allowed. The p, q, r, s, t, u, v and w parameters (Fig. 5A, right) are therefore the factors affecting K_oF_, K_oB_, K_ifF_, K_ifB_, K_isF_, K_isB_, K_is2F_ and K_is2B_, respectively, giving the drug-bound values K_oFb_, K_oBb_, K_ifFb_, K_ifBb_, K_isFb_, K_isBb_, K_is2Fb_ and K_is2Bb_. This model was then used to fit current recorded on the same neurons as before, but in the presence of tetramethrin (Fig. 5B). The values of p, q, r, s, t, u, v and w and the 4 rate-constants for drug binding and unbinding to open (K_opyrF_ and K_opyrB_) or close (K_cpyrF_ and K_cpyrB_) channel states were obtained during this second fit procedure, and were displayed on a radargraph with a log scale for visual comparison (Fig. 5C).

All data generated or analyzed during this study are included in this published article.

## Supplementary information

Supplementary information
