# Supplementary material for: Voltage-gated sodium channels from the bees Apis mellifera and Bombus terrestris are differentially modulated by pyrethroid insecticides
Source: Sci Rep. 2019 Jan 31;9:1078. doi: 10.1038/s41598-018-37278-z (PMC6355911; doi:10.1038/s41598-018-37278-z)

Supplementary Information:

Voltage-gated sodium channels from the bees *Apis mellifera* and *Bombus terrestris* are differentially modulated by pyrethroid insecticides

[1]Aklesso Kadala, [1,2]Mercédès Charreton, [3]Pierre Charnet, [3]Thierry Cens, [3]Mathieu Rousset, [4]Mohamed Chahine, [1,2]Bernard E. Vaissière and [1,2]Claude Collet*

[1]INRA, UR406 Abeilles et Environnement, 84914, Avignon, France.

[2]UMT PRADE, Protection des Abeilles dans l'Environnement, 84914, Avignon, France.

[3]CNRS, UMR 5237, Centre de Recherche de Biochimie Macromoléculaire, Université Montpellier 2, Montpellier, France.

[4]Department of Medicine, Université Laval, Quebec City, QC, Canada G1K 7P4

*To whom correspondence should be addressed:
Claude Collet
E- mail: claude.collet@inra.fr

Supplementary table 1. Amino acid changes in *Bombus* Na$_{VS}$ predicted sequence (XM_012311726) as compared with *Am* Na$_{V}$ (AMB38675).

Apis mellifera	*Bombus terrestris*<br>(amino-acid localization on Am sequence)	Localization
PQPD**P**MLEQG	P->Q (P76)	N-ter
REAAA**H**AAV**T**AADQIVK	H->V ; T->A (474 ; 478)	L-I-II
TTTTK**V**RKVSA**V**SRAANGQFTY AYQESLRKASLSLP	TATTKPRKVSAAS--------------L----- SLP<br>(534)	L-I-II
GH**I**TD**N**NQK	I->F, N->S (697, 700)	L-I-II
ED**A**MG	A->P (713)	L-I-II
TPEQ--------GDD	TPEQ**GVSTYYFPT**DD insertion 760	L-I-II
KLL**T**AVLRC	T->K (768)	L-I-II
LL**T**AVLR	T->A (775)	L-I-II
KYV**A**L**L**VFDPFVEL	A->S ; L->V (801 ; 803)	L-I-II
DME**R**VLK	R->K (837)	L-I-II
SPK**F**YFQE	F->Y (865)	DII-S3
ARF**V**KWIK	V->I (1060)	L-II-III
SDQAP----------DGIDRD	SDQAP**GEGPSNSWKE**DGIDRD insertion 1088)	L-II-III
S**A**KE**L**NQ	A->T ; L->I (1112 ; 1115)	L-II-III
**E**EGEEG**E**GV**I**GDAIIQA**E**E	E->D ; E->D ; I->L ; E->D (1221; 1227; 1230; 1238)	L-II-III
FF**I**E	I->L (1335)	DIII-S2
VM**V**SLIN**FV**A**SLC**GA**GGIQ**AF**K** **T**MR	VM**L**SLIN**LG**A**IWA**GA**ADIP**AF**RS**M R (1364)	DIII-S3-S4
**M**SR**MQ**GMR	M->V; MQ->WE (1397; 1400)	DIII-S4 *
E**T**AEIEVQTRPGE**T**G	T->S; T->A (1947;1959)	C-ter

*: new charge

Supplementary figure 1: Total sodium charges (Qtot) after ten consecutive depolarizations at 13Hz. The total cumulated sodium charge is estimated as the area under the sodium current signal recorded during a sweep of standardized duration. An example from a tetramethrin-exposed ALN is displayed in the inset (grey area). Histograms are shown with Control as reference. No species-related difference is observed with regard to tetramethrin and esfenvalerate. All pyrethroid-exposed ALNs have a Qtot significantly increased when

compared to control, regardless of the species. Statistical significances: ns= not significant; ****$p<0.0001$.

Supplementary figure 2: Sodium current paramaters extracted from the Markovian model on traces recorded in control conditions. The Markovian model was adjusted to experimental recordings in control conditions. Voltage-dependance is expressed as $A.\exp(V/k)$, where A is the value at V=0, k is the voltage dependency, and V is the membrane potential. Parameters Af, kf, Ak and kk represent the transitions from the Closed state to the Open state and parameters Aif et kif, Aib et kib represent transitions from the Open state to the Fast inactivated state (in mV^{-1}). Transitions to the Slow inactivated state are not voltage-dependant and are represented by parameters KisF, KisB (in ms^{-1}).

Supplementary figure 3: Percentages of tetramethrin- and esfenvalerate-modified channels. Mean percentage of modified voltage-gated sodium channels from *Bt* (left) and *Am* (right) along with ten depolarizations in the presence of tetramethrin (filled symbols) and esfenvalerate (open symbols). The vertical scale on the right graph is identical to the scale on the left graph. Tetramethrin modifies significantly more sodium channels than esfenvalerate in both species. Scales on Y axis are identical. Statistical significance: ****$p<0.0001$; ***$p<0.0005$; **$p<0.01$; *$p<0.05$.

Supplementary Figure 1

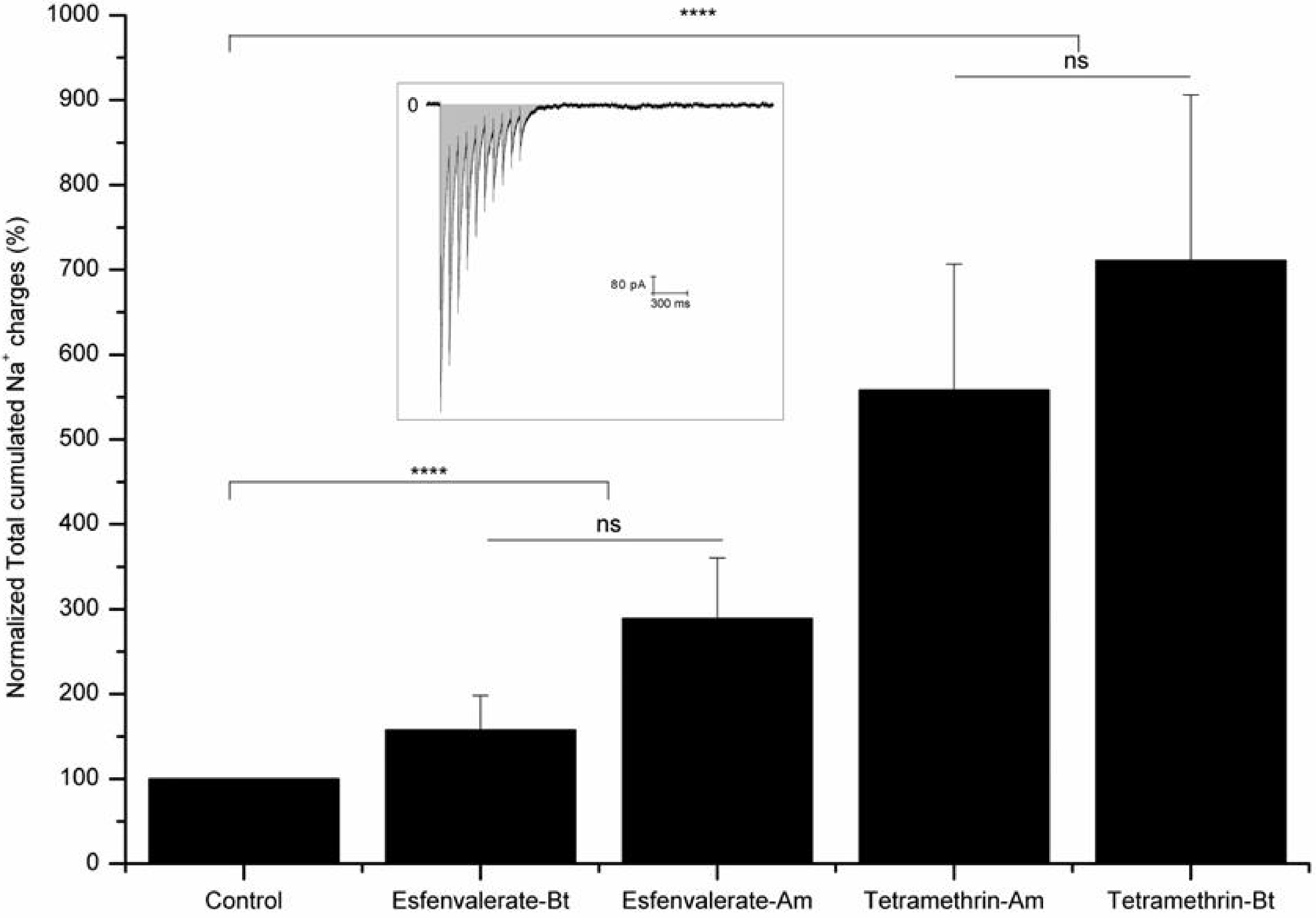

Supplementary Figure 2

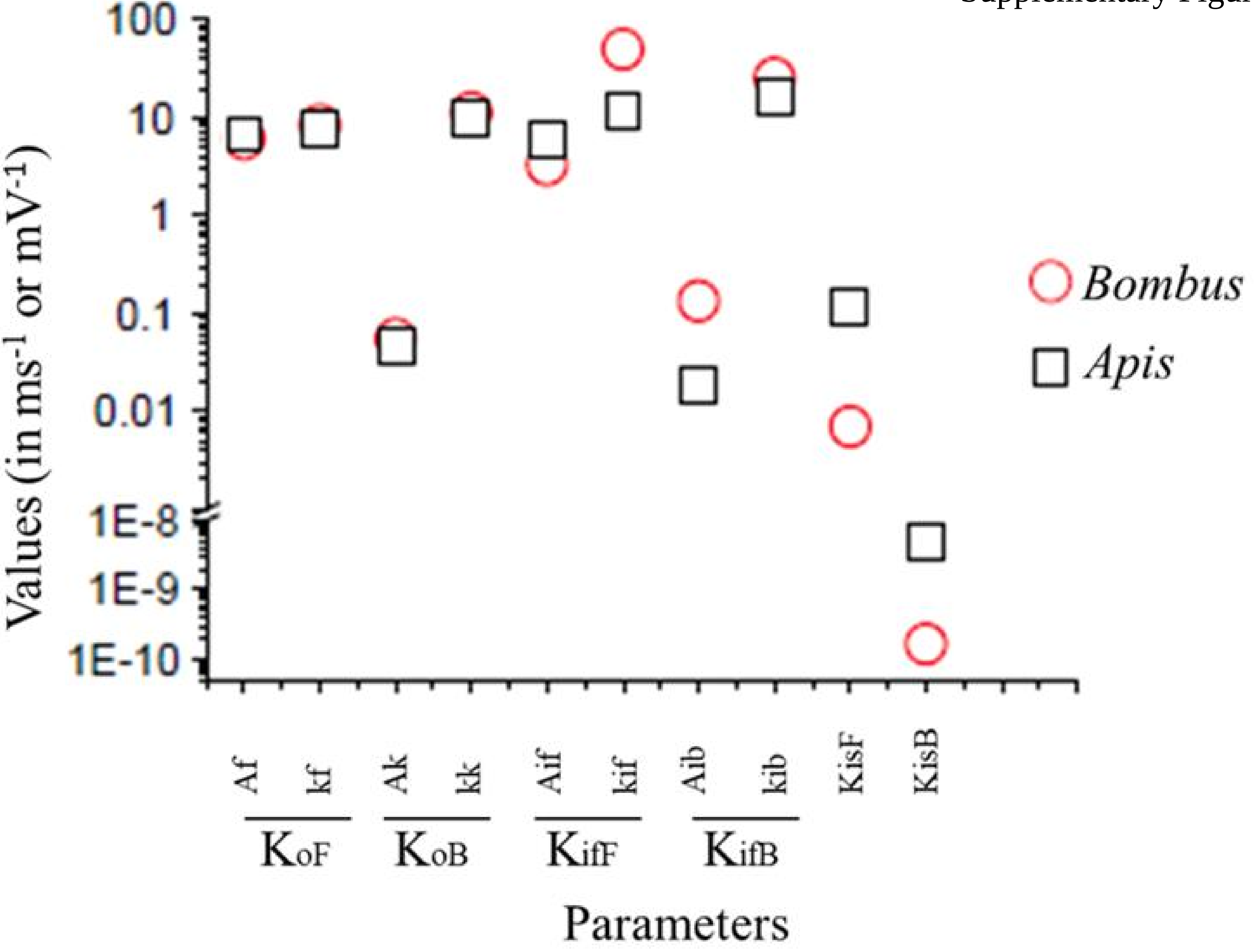

Supplementary Figure 3

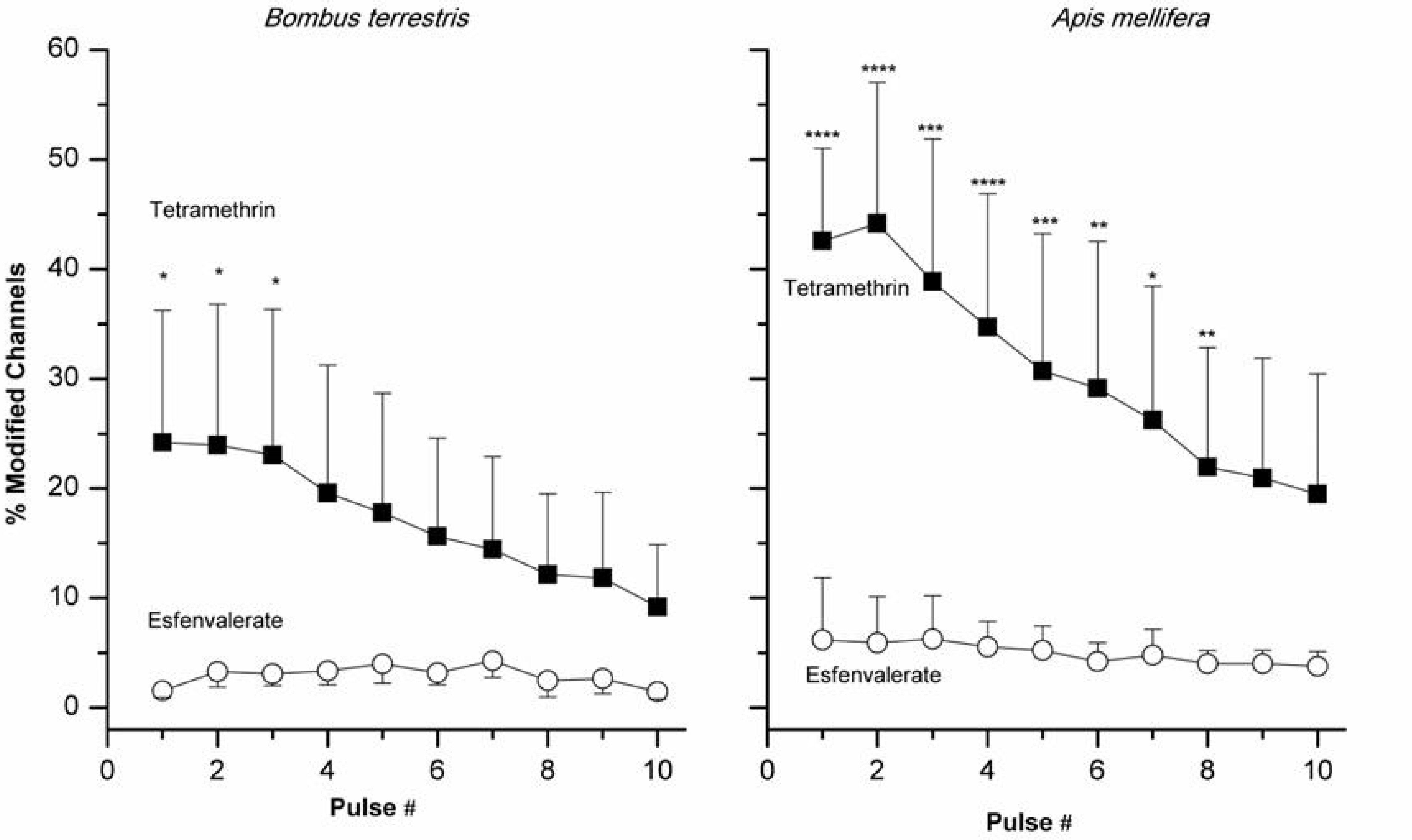

Supplement: Supplementary file 1 — Supplementary information [file 41598_2018_37278_MOESM1_ESM.pdf]
